# The Effect of Social Cohesion on Interest, Usefulness, and Ease of Use of a Driving Assistance System in Older Adults

**DOI:** 10.3390/ijerph182111412

**Published:** 2021-10-29

**Authors:** Hiroko Kamide

**Affiliations:** Institute of Innovation for Future Society, Nagoya University, Furocho, Chikusaku, Nagoya 4648601, Japan; kamide@coi.nagoya-u.ac.jp

**Keywords:** driving skills support systems, social cohesion, interest, usefulness, ease of use, older people, social cohesion

## Abstract

This study examined the relationship between social cohesion and the perceived interest in, the usefulness of, and the ease of use of an instructor-based driver assistance system in a sample of older adults. With the aging of the population, the use of technologies to support the driving skills of the elderly is expected, and it is necessary to clarify the conditions under which the elderly will be interested in these advanced technologies. Traditionally, social cohesion has been focused on as a function of instrumental and practical support in the lives of the elderly. Since social cohesion reflects the intention to help each other, it could be an opportunity to provide information on advanced driving skill techniques to older people who are becoming more difficult to drive. As an initial exploration, this study examined whether social cohesion was associated with the interest in, the usefulness of, and the ease of use of an instructor-based driver assistance system in 150 elderly people. The results showed that a greater social cohesion was significantly associated with these evaluations, and that a comprehension of the system also contributed. The possession of a license was significantly associated with interest in the program. These findings are an essential step toward the understanding of the roles of social cohesion and positive perception of advanced technology in older adults.

## 1. Introduction

Japan’s aging population is growing rapidly, and government statistics indicate that car and motorcycle collisions involving elderly people who are at fault are also on the rise. In 2009 they accounted for 15 percent of collisions, but this percentage had increased to 23 percent by 2019 [1]. The safety of elderly Japanese drivers has become an urgent issue. When older people stop driving, they are less likely to leave their homes [2], which can lead to loneliness [3]. Using public transportation to go out and socialize and partake in activities can help to reduce loneliness [4], but it is also important for older people to be able to drive safely in areas where there is a lack of sufficient public transportation. The World Health Organization [5] defines active aging as “the process of optimizing for health, participation and security in order to enhance quality of life as people age” and specifically emphasizes that active aging means staying engaged in society. 

While there is a need for the effective use of advanced technological systems to maintain and improve the driving skills of older drivers and to keep them participating in society, it will take some ingenuity to make these systems interesting and acceptable to older people. Although there have been many reports on the acceptance of technology [6,7,8,9,10,11,12], including by older adults [13,14,15,16], much of the focus is on the need, usefulness, and concerns such as costs and privacy that are directly related to technology use (see the review by Peek et al. [17]). For the last few decades, social cohesion that promotes interventions and cooperation for the mutual support and mutual prosperity within neighborhoods has been shown to be particularly important for fulfilling the lives of older people [18,19,20,21,22,23,24,25,26]. Social cohesion does not seem to be a factor that influences the acceptance of new technologies. However, since this is of practical help to the lives of the older people in neighborhood communities, it may serve as an opportunity for older people, who may have difficulty in mobility, to exchange information and interest in new assistive technologies with each other. This study focuses on the possibility of this function of social cohesion and aims to explore the relationship between social cohesion and positive subjective evaluations, such as the interest in and usefulness of newly developed driving skills support systems for older people, as a first step.

Among the theories on the acceptance of technology, the mainstream theory is the model that was proposed by Davis [11,12] and later advanced by Venkatesh et al. [7,8,9]. The key variables for these models are perceived usefulness and perceived ease of use. Higher levels of perceived usefulness and perceived ease of use, along with external factors such as attributes and experience, generally drive intentions and actual use behavior for the technology. These theories have also been applied to the acceptance of advanced driving technologies [27,28,29,30,31,32,33]. Koul and Eydgahi [34] found that these two factors were positively associated with the intention to use driverless cars. Braun et al. [35] showed that usefulness is one of the key factors in the acceptance of advanced driver assistance systems (ADAS) by older drivers. In addition to perceived usefulness and perceived ease of use, trust and attitude were also reported as important predictors [27,36]. Compared to younger adults, older adults reported a lower acceptability along with positive attitudes toward automated vehicles [37]. It has examined the extent to which their significant others (e.g., family and friends) thinking that they should use an automated road transport system influences their intention to use it [31]. However, the unity of the community needs to be considered, not just significant others, as it is important for the lives of the elderly. 

In examining the factors that support the elderly, social cohesion has received considerable attention [38]. Social cohesion includes neighborhood support resources, social participation, norms of returnability, and the social connections that bind neighborhood society [39]. This means that the members of a society are willing to cooperate with each other for survival and prosperity [40]. It also includes the trust that citizens have for each other to intervene for the common good [41]. The reason why social relationships within neighborhoods are so important for the elderly is that as they age, their environment tends to shrink to their home and other neighborhoods [42]. In other words, if the social cohesion in the neighborhood where the older adults live is sufficient, community members may try to support the mobility of the older adults by teaching them new information that supports their ability to drive. Because social cohesion functions to support the lives of older adults, experiencing low cohesion has also been reported to be associated with their poorer physical and mental health [41,43,44,45,46,47]. However, if they are more socially cohesive, older adults participate in society [48,49,50] and are able to prevent their physical disability [51]. More specifically, social cohesion is a predictor of reduced limitations in activities of daily living, such as eating and dressing, and in instrumental activities, such as shopping and dealing with bills and banking [52]. Thus, neighborhood social cohesion is associated with the health of older adults because it provides social and psychological support and increases the dissemination of health-related information [53,54].

Considering that the social environment of the neighborhood provides substantial health benefits for the elderly by providing them with social resources [55,56] and access to services [57], it is likely that high social cohesion provides opportunities for elderly people who are likely to have difficulties with mobility to come into contact with assistive technologies. Neighborhood communities may be the most important place of practice for older people [58,59], and if they are more socially cohesive, they may try to help older people’s driving abilities in many practical ways, and such cooperation and support may also help older people to learn about new technologies. While the elderly are less interested in advanced vehicle technology [60], given that social cohesion functions as a benevolent intervention for mutual prosperity, it is also possible that older people may try to become interested in or positively aware of such technologies. Identifying causal relationships is not the focus of this study but based on the above report that social cohesion influences various aspects of older adults’ lives, this study exploratively examines the potential impact of social cohesion on the interest in, usefulness of, and ease of use of the developed driving skills support system.

One of the reasons why the elderly are not open to a driving skills support system could be the difficulty in understanding advanced technology. For example, Viktorová and Šucha [61] reported that the acceptability of ADAS requires drivers to have a good understanding of the system’s possibilities and limitations. In this study, we introduced a system to older people through a video with as much detail as possible, and then had them evaluate their interest in it (details are in the methods section). It was also necessary to verify the extent to which they were able to understand the content of this video. Controlling for the degree of understanding of the video, we explored the relationship between social cohesion and perceived interest, usefulness, and ease of use. As a first step to investigate the relation between social cohesion and perception on driving skills support systems, this study used a single support system for older adults.

## 2. Materials and Methods

### 2.1. Participants and Procedure

The data used in this study were collected from older adults that were recruited by an online survey company based on our instructions regarding their age. The company adheres to the law, national guidelines and other norms, as well as the Code of the Japan Marketing Research Association. The survey company emailed its monitors who were 65 and older to invite them to participate in a survey on a driving skills support system. The monitors who received the invitation accessed the website by using a computer or tablet and participated in the survey. On the screen before the main survey started, participants answered questions about their age, gender, and whether or not they were licensed. The survey was completed in a few days. A number of 150 older adults participated in the survey (75 men, mean age = 73.55, SD = 6.27, range = 65–95), of which 90 (45 men, mean age = 72.73, SD = 5.55, range = 65–84) had a driver’s license and 60 (30 men, mean age = 74.77, SD = 7.09, range = 65–95) did not have a license. Whether or not they possessed a driver’s license was controlled for in the analysis. 

### 2.2. The Driving Skills Support System Video

There are a variety of technologies that support the driving skills of the elderly, such as robots that give driving advice [62] and systems that mediate merging between cars [62], but this study targets an instructor-based driver assistance system [63] as the first step in examining the relationship with social cohesion. This system has been advanced recently and used in social implementation experiments but it is very new to older adults. The instructor-based driver assistance system alerts the driver to the presence of a risk, such as somebody running out into the road ahead, and assists deceleration to avoid the risk. The video was made by the system developers, and they presented the system in as accurate and detailed a manner as possible. 

The video begins with a scene of a small electric car driving through a residential area, showing the view from the driver’s seat looking out. Then, after a person comes out of a side street, the system notifies the driver that it has detected a pedestrian walking by looking at her phone. In this situation, there is a risk of the pedestrian jumping out of the way, so the video shows where the system assists safe driving. Next, the system’s functions are introduced on the driving simulator. When approaching an intersection with poor visibility, the system provides braking assistance to slow down the vehicle as necessary. It is also announced that the system will not be activated if the support system determines that no assistance is needed. In addition, the driver is informed by voice and text that previous studies have shown that using this system will ensure the safety of the driver and pedestrians and improve driving skills. This video is one minute and 34 s long.

To assess comprehension of the video content, participants were asked, “How well did you understand the content of the video?” on a seven-point scale from 1: “not at all” to 7: “very well”.

### 2.3. Measures

#### 2.3.1. Social Cohesion

This study used the Japanese version of the three-item measure of social cohesion developed by Saito et al [64]. The items were: (1) “Do you think people in your community are generally trustworthy?”; (2) “Do you think that people in your community often try to be helpful to others?”; and (3) “How attached are you to the area in which you currently live?” Each item was answered on a five-point scale from 1: “totally disagree” to 5: “totally agree.” The alpha reliability was 0.85 and the averages of these items were used in the analysis.

#### 2.3.2. Interest, Usefulness, and Ease of Use

Respondents rated how they felt in the following items about the instructor-based driver assistance system they watched in the video on a seven-point scale from 1: “totally disagree” to 7: “totally agree.”

Interest included the following three items: (1) “I would like to use it,” (2) “I would like to try it myself,” and (3) “It looks interesting” (alpha reliability = 0.88). Usefulness included the following two items: (1) “I think it’s useful,” and (2) “It would be helpful,” (alpha reliability = 0.94). Ease of use included the following three items: (1) “It looks easy to use,” (2) “Anyone can use it” and (3) “It seems clumsy to use” (reversed) (alpha reliability = 0.82).

## 3. Results

Table 1 shows descriptive statistics for each variable. Because this study was an exploratory first step in examining the relationship between social cohesion and the perceptions of a driving skills support system, there were not many participants in the study. This will be discussed later as a limitation of this study.

We examined the correlation between perceived interest, usefulness, and ease of use, and the level of comprehension of the video and social cohesion. Significant correlations were shown between all variables (Table 2). Interest, usefulness, and ease of use are moderately to strongly correlated for the system that was described in the video (*r* = 0.41–0.64), and they are also moderately correlated with comprehension of the video (*r* = 0.33–0.46). The video explains how the system helps, and those who felt they understood the content better seemed to have a more positive perception of the system. Social cohesion was also shown to be positively correlated with other variables (*r* = 0.26–0.32). This means that the interest, usefulness, and ease of use of the system are also related to the social context surrounding the participants in some way. Social cohesion is also significantly correlated with video comprehension, but it is very weak (*r* = 0.18). It seems that the degree of understanding of a new system that may seem difficult for the elderly to understand is slightly related to their social relationships with others.

A multiple linear regression using forced entry was calculated to predict interest based on age, gender (1 = man, 2 = woman), license (1 = non-licensed, 2 = licensed), video comprehension, and social cohesion. A significant regression equation was found (F(5144) = 14.72, *p* < 0.001), with an adjusted R^2^ of 0.32. Having a driver’s license promoted interest in the instructor-based driver assistance system (β = 0.22, *p* < 0.01). A higher understanding of the video (β = 0.39, *p* < 0.01) and a higher social cohesion (β = 0.23, *p* < 0.01) were also associated with a stronger interest in the system.

A multiple linear regression using forced entry was also calculated to predict usefulness based on age, gender, license, video comprehension, and social cohesion. A significant regression equation was found (F(5144) = 8.22, *p* < 0.001), with an adjusted R^2^ of 0.20. No effect of licensure was shown on the perception of usefulness, and a higher understanding of the video (β = 0.37, *p* < 0.01) and social cohesion (β = 0.21, *p* = 0.01) were associated with higher usefulness.

Then, a multiple linear regression using forced entry was calculated to predict ease of use based on age, gender, license, video comprehension, and social cohesion. A significant regression equation was found (F(5144) = 6.05, *p* < 0.001), with an adjusted R^2^ of 0.15. It was shown that a higher video comprehension (β = 0.28, *p* < 0.01) and social cohesion (β = 0.21, *p* = 0.01) were associated with a higher perceived ease of use. In these analyses, the VIF value was 1.03–1.06, which did not indicate multicollinearity. The summary of regression analysis was shown in Table 3, Table 4 and Table 5.

Social cohesion was shown to be a positively significant predictor of perceived interest, usefulness, and ease of use of the instructor-based driver assistance system. Comprehension of the video was also a significant predictor of all of these, and license ownership was significantly related to an interest in the system. Age and gender were not significantly associated with a perceived interest in, usefulness of, or ease of use of the system.

## 4. Discussion

This study explored the relationship between social cohesion of older adults and their interest in, usefulness of, and perceived ease of use of systems that support their driving skills. Results showed that social cohesion significantly correlated with interest, usefulness, and ease of use about the instructor-based driver assistance system. The results of multiple regression analyses showed that social cohesion was a significant predictor of interest, usefulness, and ease of use along with the level of understanding of the video that showed how the system works. Possession of a license was a significant predictor of interest in the system. While previous studies have discussed factors that are directly related to technology in terms of the acceptance of technology and the social impact of significant others, this study showed that the social environment surrounding the elderly is one possible source of motivation for their interest in and positive perception of technology.

Social cohesion has been reported to be a source of physical and mental health in the elderly [22,38,41,45,46,47,51,65,66,67]. The reason for this is that having social connections has practical implications for the elderly [58,59] and provides them with opportunities to gain social and material resources [55,56,57]. Mobility difficulties increase as we age; therefore, the elderly may need assistance with mobility, as reduced mobility in the elderly reduces their quality of life [68]. Given the fact that many elderly people are now concerned about motor vehicle collisions when they drive themselves [1], they may feel some need for a means to help them maintain and improve their driving skills. According to the results of this study, social cohesion may function to promote older adults’ interest in and positive perceptions of advanced driving skills. If they have a stronger intention to intervene for mutual prosperity, they might share that information with an elderly person who might need information about a new driver assistance technology. Or, if the neighbors are considerate of each other, then older people may be more open to technologies that can ensure the safety of one another. The mechanisms by which social cohesion is associated with positive perceptions of assistive systems will be explored in future research.

It was shown that social cohesion was associated with positive perceptions of the instructor-based driver assistance system, but this study is still exploratory and in its initial stages. While previous evidence has shown that subjective perceptions of cohesion are important [22,43,65,69,70], it has also been reported that neighborhood-level social cohesion is not consistent among residents of the same neighborhood [71,72]. A neighborhood is defined as a contiguous, physical area where many residents form a large part of their daily interpersonal network [46], and may be limited to a radius of 600 m or less within walking distance [73]. Since this study was simply presented as “your community” or the area in which you live, it needs to be examined for further confirmation with a detailed physical and psychological definition of the neighborhood. 

Relatedly, the relationship between social cohesion and economic status has been noted. People living in areas of high economic deprivation report lower social cohesion than those living in areas of low economic deprivation [74]. For the elderly, the higher the level of poverty in a neighborhood, the lower the perception of social cohesion and the worse the mental health [45]. The present study showed an association between social cohesion and perceptions of the instructor-based driver assistance system, and this relationship may be influenced by socioeconomic status. Since such systems are usually expensive, it may be that economically affluent people simply have a higher social cohesion and are more interested in this system at the same time. On the other hand, social cohesion has also been reported to affect individual health by mitigating the effects of poverty and inequality [75,76]. Therefore, in order to understand the relationship between perceptions of advanced technology and social cohesion in more detail, it is necessary to consider the relationship with socioeconomic status and poverty as well.

It is not surprising that having a license is associated with interest in the instructor-based driver assistance system. It is possible that social cohesion may be associated with holding a driver’s license, although this study did not examine it because it focused on the effect of social cohesion on positive perceptions of the system. Since social cohesion is related to social participation and close help within the community, older people may need to drive in order to participate in such activities. In addition to examining social cohesion and driver’s license ownership independently, the impact of their interaction effect needs to be carefully examined in the future. Although the license variable was not associated with usefulness or ease of use, this may be due to the fact that the system is new to both licensed and unlicensed seniors, and they have not yet used it. Considering that the experience of actually using the system influences the perception of the technology [7], changes over time will need to be considered in the future. In addition, while the technology acceptance model includes the fact that the perception of usefulness and ease of use eventually influences the intention and behavior to use the technology [6,7,8,10,11,12,61], in this study, we examined the relationship with only the factors that preceded use. This is because we focused first on whether or not social cohesion is associated with the preliminary stages of interest and positive evaluation, rather than the stage where the system is still in actual use for the elderly. As technologies to support mobility for the elderly will continue to be developed, it will be necessary to consider the intentions and behaviors of use in the social context when these technologies are implemented in society. 

There are other major limitations of this study. First, the number of elderly people included in the study was very limited. The results of this study and the discussion above presented only a few of the possibilities for the relationship between social cohesion and evaluation of driver assistance systems among the elderly. This study is really a rudimentary attempt to explore whether or not these factors are related, and the extent of this possibility needs to be explored in larger samples in the future. Our sample consisted of older adults who were registered as monitors with an online survey company and were familiar with the Internet. It is possible that older people are familiar with new technology, or that there is a bias that caused this small sample to be more responsive to the system. While this study may have shown one possible explanation for social cohesion and perceptions of support systems, it remains far from generalizable, and further confirmation is essential. In addition, since this research was the first step, only one form of driver assistance technology was targeted. Other systems are currently being developed to support the driving skills of the elderly, and it remains to be seen whether the results of this study can be applied to those technologies. It is necessary to further examine how these technologies will be accepted by the elderly in a social context, and to take into account their technological features. A more serious limitation of the stimuli is that the video used in this study only presented the positive effects of the system. In reality, however, it is possible that there are disadvantages and drawbacks that can arise from using the system, depending on the person and the situation. The video used in this study did not include any explanation of such negative aspects. One of the reasons for the relatively high level of understanding of the video was that only the positive aspects were presented in a short period of time, which did not make the viewers question and think about them. It is important for explanations of new systems and technologies to include both advantages and disadvantages and to provide details of existing facts and possible situations, and it is essential to present them more clearly in the future research. It was stated in the introduction that social cohesion may be a factor for older people to be interested in new devices, but this study is cross-sectional, and causality has not been identified. As already mentioned, there is a need to examine changes over time with regard to perceptions and the acceptance of technology. Longitudinal studies, such as comparisons before and after the use of technology, are needed in the future.

## 5. Conclusions

This study examined the association of social cohesion with perceived interest in, usefulness of, and ease of use of the instructor-based driver assistance system in a sample of older adults. In exploring these associations, we also examined license ownership and understanding of the system’s instructions. The results showed that greater social cohesion was significantly associated with interest in, usefulness of, and ease of use of the system, and that level of understanding was also associated with these. Possession of a license was significantly associated with interest. Further studies are needed with other driver assistance systems, larger samples, and economic status taken into account.

## Figures and Tables

**Table 1:** Descriptive statistics for each variable (*n* = 150).

	Min	Max	Mean	SD
Age	65	95	73.55	6.27
Scial cohesion	1	5	3.58	0.70
Video comprehension	1	7	5.83	1.26
Interest	1	7	4.47	1.66
Usefulness	1	7	4.94	1.27
Ease of use	1	6	4.30	0.68

**Table 2:** Correlation among interest, usefulness, ease of use, video comprehension, and social cohesion.

Variables	Interest	Usefulness	Ease of Use	Video Comprehension
Interest	-			
Usefulness	0.64 **	-		
Ease of use	0.41 **	0.62 **	-	
Video comprehension	0.46 **	0.41 **	0.33 **	-
Social cohesion	0.32 **	0.27 **	0.26 **	0.18 *

Note: ** *p* < *0*.01, * *p* < *0*.05.

**Table 3:** Result of regression analysis for interest (*n* = 150).

Variables	B	SE B	β	t	*p*	VIF
age	−0.03	0.02	−0.10	−1.41	0.16	1.05
gender	−0.34	0.23	−0.10	−1.50	0.14	1.03
Licence	0.73	0.23	0.22	3.13	0.00	1.05
Video comprehension	0.51	0.09	0.39	5.57	0.00	1.06
Social cohesion	0.55	0.16	0.23	3.37	0.00	1.05

Note: β = standardized coefficient of regression of the predictors.

**Table 4:** Result of regression analysis for usefulness (*n* = 150).

Variables	B	SE B	β	t	*p*	VIF
age	−0.03	0.02	−0.14	−1.84	0.07	1.05
gender	−0.10	0.19	−0.04	−0.54	0.59	1.03
Licence	−0.04	0.20	−0.02	−0.22	0.83	1.05
Video comprehension	0.37	0.08	0.37	4.85	0.00	1.06
Social cohesion	0.37	0.14	0.21	2.72	0.01	1.05

Note: β = standardized coefficient of regression of the predictors.

**Table 5:** Result of regression analysis for ease of use (*n* = 150).

Variables	B	SE B	β	t	*p*	VIF
age	−0.01	0.01	−0.11	−1.46	0.15	1.05
gender	0.05	0.10	0.04	0.50	0.62	1.03
Licence	0.07	0.11	0.05	0.67	0.50	1.05
Video comprehension	0.15	0.04	0.28	3.59	0.00	1.06
Social cohesion	0.20	0.08	0.21	2.65	0.01	1.05

Note: β = standardized coefficient of regression of the predictors.

## Data Availability

To protect privacy, the data is confidential and is not available.
